# Image Reconstruction Using a Mixture Score Function (MSF)

**Published:** 2024-01-09

**Authors:** Wenxiang Cong, Wenjun Xia, Ge Wang

**Affiliations:** Biomedical Imaging Center, Center for Biotechnology and Interdisciplinary Studies, Department of Biomedical Engineering, Rensselaer Polytechnic Institute, Troy, NY 12180

**Keywords:** Computed tomography (CT), radiation dose reduction, image reconstruction, maximum a posteriori (MAP) estimation, score function, deep learning

## Abstract

Computed tomography (CT) reconstructs volumetric images using X-ray projection data acquired from multiple angles around an object. For low-dose or sparse-view scans, the classic image reconstruction algorithms often produce severe noise and artifacts. To address this issue, we develop an iterative image reconstruction method based on maximum a posteriori (MAP) estimation. In the Bayesian framework, the gradient of logarithmic probability density distribution of the image, i.e., the score function, plays a crucial role, contributing to the process of image reconstruction. By leveraging Gaussian mixture model, we derive a novel score matching formula to establish a mixture score function (MSF) through deep learning. The MSF-based iterative reconstruction algorithm significantly improves image reconstruction quality. The convergence of the MSF iterative reconstruction algorithm is first proven through mathematical analysis. Then, the performance of the MSF reconstruction method is evaluated on both public medical image datasets and clinical raw CT dataset. Our results show that our proposed method outperforms state-of-the-art reconstruction methods in terms of accuracy and generalizability.

X-ray computed tomography (CT) is the most popular imaging modality used in various fields, including medical imaging, homeland security and industrial applications [1]. Filtered backprojection (FBP) is an analytical method for tomographic image reconstruction [2], and often produces noise and artifacts in low-dose or sparse-view CT scans [3]. To address this challenge, iterative reconstruction methods were developed by incorporating the statistical distribution of photons and prior image characteristics especially sparsity [3, 4]. Compressed sensing (CS)-based reconstruction methods convert images into sparse data using transformation techniques such total variation (TV) minimization, and perform image reconstruction through sparsity regularization. However, CS-based image reconstruction tends to over smoothen textures and subtle details in images. To better utilize the low dimensional characteristics of the image patch manifold, the low-dimensional manifold-based reconstruction approach was developed to minimize the data fidelity term and the dimensionality of the image patch manifold [5, 6]. However, these assumptions did not reflect actual image structures accurately and comprehensively, especially for sophisticated biomedical images.

Emerging deep learning is a powerful approach to perform various types of uncertainty estimation and data modeling [7]. Supervised learning was applied to establish deep convolution network model for image denoising and deburring [8], and perform image reconstruction using unrolling iterative optimization schemes by replacing the conventional regularization terms with trained convolutional neural networks (CNNs) [9, 10]. Supervised learning requires a labeled dataset paired with input data, which usually unavailable in practice. Recently, diffusion models or score matching models have attracted widespread attention for image generation [11] and image reconstruction [12, 13]. In an unsupervised fashion, the score matching method uses a neural network to learn the gradient of the logarithmic probability density function of images from a training dataset. Significantly differ from conventional regularization methods that assume images to be piecewise constants, sparsity, low rank, and low-dimensional, the score function is a much more accurate representation of the distribution of underlying images.

In this paper, we develop a score function-based algorithm for CT image reconstruction. X-ray projection data can be modeled as a Poisson distribution, which allows reconstructing an image using maximum a posteriori (MAP) estimation. In the Bayesian statistics framework, the score function plays a crucial role, contributing to the process of image reconstruction. By leveraging Gaussian mixture to characterize discrepancies between a reconstructed image and the real image, here we derive a novel score matching formula to generate a mixture score function (MSF) through deep learning. The score function as an optimal prior is then integrated into the iteration process of image reconstruction. The convergence of the iterative reconstruction algorithm is proven through mathematical analysis. The performance of proposed image reconstruction method is evaluated on both public medical image datasets and clinical raw datasets, demonstrating its superiority over representative state-of-the-art image reconstruction methods in terms of accuracy and generalizability.

## Results

Based on the CT image benchmark used in the NIH-AAPM-Mayo Clinic low-dose CT Challenge, the deep learning method based on the score matching formula Eq. (16) was used to establish mixture scoring function (MSF) described in the Methods Section. Using the MSF, we conducted extensive experiments on public medical image datasets and clinical CT raw datasets to evaluate the performance of the MSF reconstruction method by comparing with representative image reconstruction methods, namely the classic filtered backprojection (FBP) [1], simultaneous algebraic reconstruction technique (SART) with total variation (TV) [14], and state-of-the-art score function-based image reconstruction [12, 13].

### Phantoms

We evaluated the performance of the MSF reconstruction method in the three settings: low-dose, few-view, and normal-dose full scan using 100 CT slices from different patients as numerical image phantoms. A representative slice was presented as an example to show image reconstruction performance.

#### Low-dose setting:

**Figure 1.**
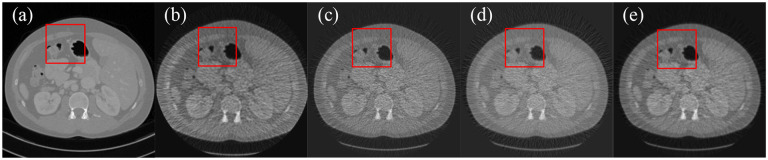

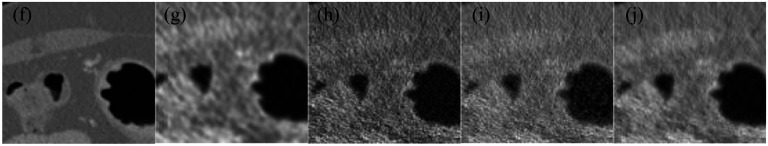
Comparison of the reconstructed images from low dose projection data. (a) The ground truth image, (b) the image reconstructed using FBP, (c) the image reconstructed by SART with total variation, (d) the image reconstructed by the current cutting-edge score function-based method, (e) the image reconstructed by our MSF reconstruction method, and (f–j) the enlarged images in the red rectangle in (a–e), respectively.

CT images came from the NIH-AAPM-Mayo Clinic CT Grand Challenge. The imaging protocol had a scanning a radius of 54.1 cm. The distance from the light source to the detector was 94.9cm. There were 888 detector elements with 0.1024cm pitch equiangularly distributed along a curvilinear array. X-ray imaging was simulated with 10^4^ photons per path using a distance-driven algorithm to generate fan-beam projections. A total of 360 projection views were uniformly acquired over a 360-degree range. The projection datasets were corrupted by Poisson noise to simulate real X-ray imaging experiments. We performed fan-beam image reconstruction from low-dose projections using FBP, SART with total variation regularization, score function-based method, and our MSF reconstruction method, respectively. Figure 1 displays the reconstructed images and zoom-in image patches for comparison.

#### Few-view setting:

**Figure 2.**
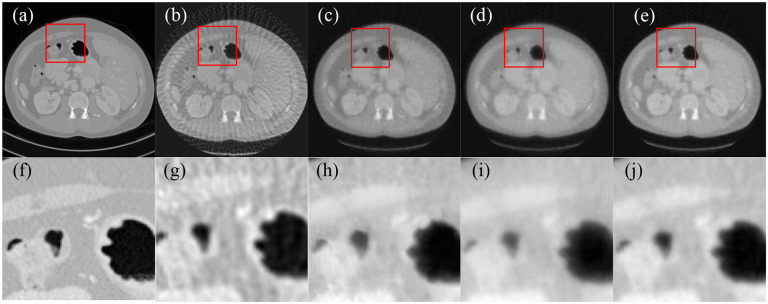
Comparison of the reconstructed images from few-view projection data. (a) The ground truth image, (b) the image reconstructed using FBP, (c) the image reconstructed by SART with total variation, (d) the image reconstructed by the score function-based method, (e) the image reconstructed by the MSF reconstruction method, and (f–j) the enlarged image in the red rectangle in (a–e), respectively.

Imaging geometry was the same as in the low-dose case. X-ray imaging was simulated with 10^5^ photons per path using a distance-driven algorithm to generate fan-beam projections. A total of 80 projection views were uniformly acquired over a 360-degree range to generate a few-view projection data. The projection data were corrupted by Poisson noise to simulate real x-ray imaging experiments. Figure 2 presented the images reconstructed from few-view projection data using FBP, SART with total variation regularization, the score function-based method, and the MSF reconstruction method, respectively.

#### Normal-dose full-scan setting:

**Figure 3.**
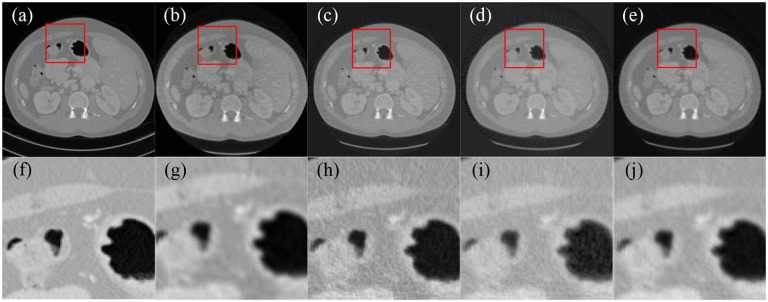
Comparison of the reconstructed images from normal dose projection data. (a) The ground truth image, (b) the image reconstructed using FBP, (c) the image reconstructed by SART with total variation, (d) the image reconstructed by score function-based method, (e) the image reconstructed by MSF reconstruction method, and (f–j) enlarged image for red rectangle in images (a–e), respectively..

Imaging geometry remained the same. X-ray imaging was still simulated with 10^5^ photons per path using a distance-driven algorithm. A total of 360 projection views were uniformly acquired over a 360-degree range. The projection data were corrupted by Poisson noise. Figure 3 presented representative images reconstructed from normal-dose full scan projection data using FBP, SART with total variation regularization, the score function-based method, and the MSF reconstruction method, respectively.

Image reconstructions were performed for 100 CT slice phantoms, and reconstructed image qualities were quantitatively evaluated using peak signal-to-noise ratio (PSNR) and structural similarity index (SSIM) metrics. As a result, the MSF reconstruction method achieved higher average PSNR value and SSIM index than the other reconstruction methods, as shown in Table 1. Intuitively, by comparing reconstructed images in Figures 1–3, it can be observed that the image reconstructed by the FBP algorithm is noisier than the image reconstructed by other reconstruction methods in the low-dose and few-view scenarios, while the MSF reconstruction method outperforms the other reconstruction methods in all three scenarios, and well preserves structural information, and achieves notable denoising and deblurring effects.

### Clinical data

Comparing FBP, SART with total variation regularization, and the score function-based reconstruction methods, the performance of MSF reconstruction method was evaluated on clinical raw datasets as follows.

#### Clinical raw data from Siemens scanner:

**Figure 4.**
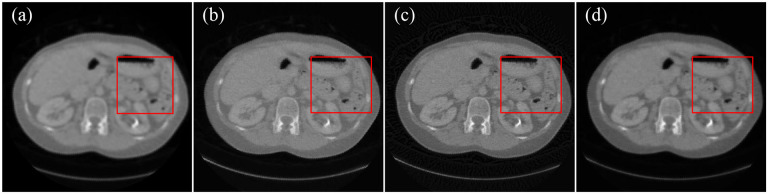

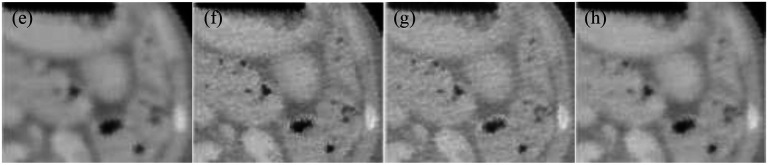
Comparison of CT image reconstructions from the Siemens scanner raw data of 576 projection views. (a) The image reconstructed using FBP, (b) the image reconstructed by SART with total variation, (c) the image reconstructed by score function-based method, (d) the image reconstructed by MSF reconstruction method, and (e–h) the enlarged image in the red rectangle in images (a–d), respectively.

NIH-AAPM-Mayo Clinic Low-dose CT Grand Challenge contained 10 anonymized patient normal dose abdominal CT images. The dataset was acquired by the Siemens SOMATOM Definition Flash CT system with voltage 120 KVp and 200 mAs. The helical raw data were converted to flat detector fan-beam projections. X-ray imaging had a scanning radius of 59.5cm. The distance from the light source to the detector was 108.56cm. There were 736 detector elements with 0.1258cm pitch, uniformly distributed along a linear array. Over a 360-degree angular range, 576 projection views were uniformly acquired for image reconstruction. Figure 4 presented the images reconstructed from fan-beam data using FBP, SART with total variation regularization, the score function-based method, and the MSF reconstruction method, respectively.

#### Clinical raw data from GE scanner:

**Figure 5.**
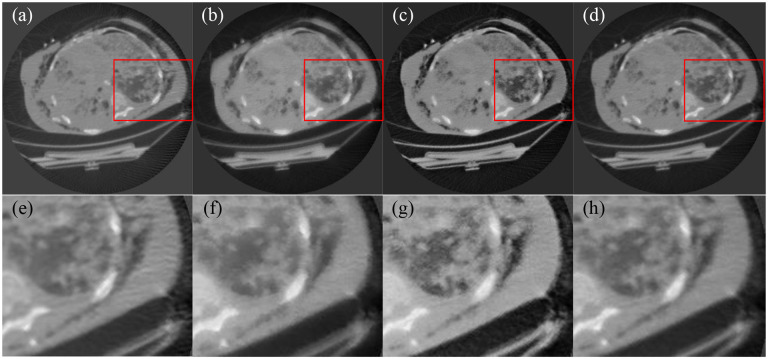
Comparison of the CT image reconstructions from GE scanner raw data of 246 projection views. (a) The image reconstructed using FBP, (b) the image reconstructed by SART with total variation, (c) the image reconstructed by score function-based method, (d) the image reconstructed by MSF reconstruction method, and (e–h) the enlarged image in the red rectangle in images (a–d), respectively.

Clinical raw data were acquired from GE CT scanner for the evaluation of the image reconstruction methods. In the scanner, X-ray imaging had a scanning radius of 54.1 cm. The distance from source to detector was 94.9cm. There were 888 detector elements with 0.1024cm pitch equiangularly distributed along a curvilinear array. There were 246 projections were uniformly acquired over a 360-degree angular range. Figure 5 presented the images reconstructed from raw data using FBP, SART with total variation regularization, the score function-based method, and the MSF reconstruction method.

From the image reconstruction results from the clinical raw data, FBP, SART with total variation regularization, and score function-based methods still exhibit blurry boundaries and unremoved noise, while our MSF reconstruction method is quite robust in denoising and deblurring.

## Discussion

In this study, we model the X-ray projection data as the Poisson distribution, and reconstruct image via maximum a posteriori (MAP) estimation. Under the MAP estimation framework, the CT images can be reconstructed by minimizing the objective function incorporated with a photon statistical model and an image prior. The image reconstruction process can be performed through gradient-based optimization, which operates in an iterative procedure. The probability density distribution as image prior knowledge plays a crucial role in the MAP estimation. The score function, the gradient of the logarithmic probability density function, contributes to the process of image reconstruction. It incorporates complete statistical knowledge and serves as the key element in this iterative process, contributing to refine the reconstructed image in the each iteration. By leveraging the Gaussian mixture model to characterize noise distributions between a reconstructed image and the real image, we have developed a novel score matching formula to learn a mixture score function from a CT image dataset. The image reconstruction has a complex relationship between the reconstructed images and the true images. Such Gaussian mixtures can accurately describe image noise distributions while inheriting the advantages of the Gaussian distribution. Importantly, the learned mixture scoring function can be used for image reconstruction on raw projection data produced by scanners from different vendors, showing excellent generalizability and stability. The proposed mixture score matching formula can also be applied to image denoising, image deblurring, and image generation to improve image quality.

Popular compressive sensing-based image reconstruction methods rely on regularization methods, which assume that images can be transformed into sparse, low-rank, or low-dimensional counterparts using transformation methods, such as total variation, wavelet transform, and Fourier transform. However, these assumptions do not always accurately reflect structures of actual images, especially for sophisticated biomedical images. In contrast, the score function approach learns directly from the data distribution, and provides much more realistic, flexible, and datadriven prior information for image reconstruction, allowing a superior representation of the underlying images.

In summary, we have proposed a novel score-matching formula to learn a mixture score function from a CT image dataset. The mixture score function as an image prior has well been integrated into the iteration process of image reconstruction in the MAP estimation framework. The convergence of the MSF iterative reconstruction algorithm has been demonstrated through mathematical analysis. The performance of MSF image reconstruction method has been evaluated on both public medical image datasets and clinical raw datasets. By comparing with the wellknown image reconstruction techniques such as the filtered backprojection (FBP), simultaneous algebraic reconstruction technique (SART) with total variation (TV) regularization, and the score function-based reconstruction method, the quantitative evaluation results has shown that our proposed MSF reconstruction method can achieve higher quality images in terms of PSNR and SSIM metric on diverse dataset. For clinical data, the MSF image reconstruction approach has achieved significantly denoising and deblurring effects. Further systematic evaluation and studies are ongoing to translate this new approach for clinical CT tasks.

## Methods

Maximum a Posteriori (MAP) estimation is a statistical technique used to maximize the probability distribution of a dataset by incorporating image prior knowledge.

### Image reconstruction approach

In X-ray imaging, the number of X-ray photons recorded by a detector element is a random variable ξ, which can be modeled as the Poisson distribution [2]:

(1) $$p\left(\xi =y_{i}\right)=\frac{{\left({\overset{‾}{y}}_{i}\right)}^{y_{i}}}{y_{i}!}exp\;\left(-{\overset{‾}{y}}_{i}\right),$$

where ${\overset{‾}{y}}_{i}$ is the expectation value of recorded X-ray photons along a path l from the X-ray source to the $i^{\text{th}\;}$ detector element, and obeys the Beer-Lambert law:

(2) $${\overset{‾}{y}}_{i}=n_{i}exp\;\left(-\int_{l}\;\;\mu (\vec{r})dl\right),$$

where $n_{i}$ is the number of X-ray photons recorded by the $i^{\text{th}\;}$ detector element in the blank scan (without any object in the beam path), and μ(r) is the linear attenuation coefficient distribution within an object to be reconstructed. For the numerical implementation, Eq. (2) is discretized as,

(3) $${\overset{‾}{y}}_{i}=n_{i}exp\;\left(-A_{i}\mu \right),$$

where μ is a vector of pixel values in the linear attenuation coefficient image, and $A_{i}$ is weighting coefficients of the pixel values along the $i^{\text{th}\;}$ beam path. Assuming that measurements are independent, the likelihood function of the X-ray projection data can be obtained by

(4) $$p(Y|\mu )=\prod_{i=1}^{m}\;\frac{{\left({\overset{‾}{y}}_{i}\right)}^{y_{i}}}{y_{i}!}exp\;\left(-{\overset{‾}{y}}_{i}\right),$$

where $Y={\left(y_{1},y_{2},\cdots ,y_{m}\right)}^{T}$ is the number of photons measured by detectors, and m is the total number of X-ray detectors. Based on the Bayesian theorem: p(μ∣Y)p(Y)=p(Y∣μ)p(μ), the image reconstruction can be performed using the maximum a posteriori (MAP) estimation, which is equivalent to the following minimization problem [15]:

(5) $$\mu_{min}=\underset{\mu }{arg\;min}\left(\sum_{i=1}^{m}\;\;\left[{\overset{‾}{y}}_{i}-y_{i}log\;\left({\overset{‾}{y}}_{i}\right)\right]-log\;p(\mu )\right),$$

where logp(μ) is the logarithmic probability density of an attenuation image, which expresses the prior knowledge about the underlying images in a specific application domain. Combining Eqs. (3)–(5), we have

(6) $$\mu_{min}=\underset{\mu }{arg\;min}\left(\sum_{i=1}^{m}\;\;\left[n_{i}exp\;\left(-A_{i}\mu \right)+y_{i}A_{i}\mu \right]-log\;p(\mu )\right),$$

Applying the second-order Taylor approximation, Eq. (6) can be simplified to the following quadratic optimization problem [4]:

(7) $$\mu_{\text{min }}=\underset{\mu }{arg\;min}\left[\frac{1}{2}(A\mu -b{)}^{T}D(A\mu -b)-log\;p(\mu )\right]$$

where A is the m×n system matrix composed of the row vectors $A_{1},A_{2},\cdots ,A_{m}$, and D is the diagonal matrix in the form $\text{diag}\;\left(y_{1},y_{2},\cdots ,y_{m}\right)$. The optimization problem defined by Eq. (7) can be solved using the gradient-based method iteratively:

(8) $$\mu^{k+1}=\mu^{k}-\left(\omega A^{T}D\left(A\mu^{k}-b\right)-\sigma \nabla log\;p\left(\mu^{k}\right)\right),\;k=1,2,\cdots$$

where ∇logp(μ) is the score function of the probability density distribution with respect to the current image, and ω and σ are parameters for trade-offs between data fidelity and sample plausibility.

### Convergence of score-function-based image reconstruction

For the convergent analysis of the iteration scheme Eq. (8), we assume that A is a m×n system matrix of rank n. The probability density function p(μ) is assumed to be sufficiently smooth, and the Hessian matrix $\nabla^{2}log\;p(\mu )$ of the logarithmic probability density function has bounded eigenvalues [16, 17], denoted by $\left|h_{i}\right|<C,\;i=1,2,\cdots ,n$. Based on these assumptions, we have the following Lemma for the convergence of the iteration scheme Eq. (8).

**Lemma 1:** The iteration scheme Eq. (8) is convergent.

**Proof:** Based on the iteration procedure Eq. (8), we obtain

(9) $$\mu^{k+1}-\mu^{k}=\left(I-\omega A^{T}DA\right)\left(\mu^{k}-\mu^{k-1}\right)+\sigma \left[\nabla log\;p\left(\mu^{k}\right)-\nabla log\;p\left(\mu^{k-1}\right)\right],$$

Based on the mean value theorem of a multivariate function, there exists a vector ξ such that,

(10) $$log\;p\left(\mu^{k}\right)-log\;p\left(\mu^{k-1}\right)=\nabla log\;p(\xi )\left(\mu^{k}-\mu^{k-1}\right).$$

From Eqs. (9–10), we obtain

(11) $$\mu^{k+1}-\mu^{k}=\left(I-\omega A^{T}DA+\sigma \nabla^{2}log\;p(\xi )\right)\left(\mu^{k}-\mu^{k-1}\right),$$

where $\nabla^{2}log\;p(\xi )$ is the Hessian matrix of the logarithmic probability density function and is symmetric. Since $(Ax{)}^{T}DAx=\sum_{i=1}^{m}\;y_{i}{\left(A_{i}\cdot x\right)}^{2}>0$ for any nonzero vector $x\in R^{n}$, the matrix $A^{T}DA$ is positive definite, denoting its smallest and largest eigenvalues as $\lambda_{min}$ and $\lambda_{max}$ respectively. From Eq. (11), it is easy to find that by choosing the parameters ω and σ to satisfy that $0<\sigma <\omega r_{\text{min }}/C$ and $\sigma C/r_{\text{min }}<\omega <(2-\sigma C)/r_{\text{max }}$, there exist a positive constant 0<q<1 such that $∥\mu^{k+1}-\mu^{k}∥\leq q∥\mu^{k}-\mu^{k-1}∥$, and $∥\mu^{k+1}-\mu^{k}∥\leq q^{k}∥\mu^{1}-\mu^{0}∥$. Hence, according to the Cauchy convergence criterion, the image sequence $\left\{\mu^{k}|k=0,1,\cdots \right\}$ must converge.

### Score function estimation

The score function is the gradient of the logarithmic probability density with respect to a current image, i.e., $\nabla log\;p_{\text{data }}(x)$. The unknown score function in Eq. (8) must be estimated in advance to perform the image reconstruction. If a known dataset sampled from a data distribution $p_{\text{data }}(x)$ is available, the score function $\nabla log\;p_{\text{data }}(x)$ can be estimated using the score matching method, which is introduced by Hyvärinen (2005) [18]. The score matching method is to train a neural network model $s_{\theta }(x)$ by optimizing model parameters θ to best match the score function $\nabla log\;p_{\text{data }}(x)$. The task can be performed by minimizing the following objective function:

(12) $$E_{p_{\text{data}}}\left[{∥s_{\theta }(x)-\nabla log\;p_{\text{data}\;}(x)∥}_{2}^{2}\right].$$

It has been demonstrated that the above objective function is equivalent to the following expectation [19]:

(13) $$E_{p(\tilde{x}|x)p_{\text{data}}(x)}\left[{∥s_{\theta }(\tilde{x})-\nabla_{\tilde{\chi }}log\;p(\tilde{x}|x)∥}_{2}^{2}\right].$$

The data x is perturbed with a noise distribution $p(\tilde{x}|x)$, which can be accurately modeled by the Gaussian mixture,

(14) $$p(\tilde{x}|x)=\int \;p(\sigma )G\left(\tilde{x};x,\sigma^{2}I\right)d\sigma ,$$

where $G\left(\tilde{x};x,\sigma^{2}I\right)$ is the Gaussian distribution with the variance $\sigma^{2}$. The Gaussian mixture represents complex noise effectively in real scenarios [20]. From Eq. (14), the gradient of logarithmic noise distribution can be calculated,

(15) $$\left\{\begin{array}{c} \nabla_{\tilde{x}}log\;p(\tilde{x}|x)=\int \;\lambda (\sigma ,\tilde{x}-x)\frac{x-\tilde{x}}{\sigma^{2}}d\sigma \\ \lambda (\sigma ,\tilde{x}-x)=\frac{p(\sigma )G\left(\tilde{x};x,\sigma^{2}I\right)}{\int \;p(\sigma )G\left(\tilde{x};x,\sigma^{2}I\right)d\sigma } \end{array}\right.$$

From Eqs. (13) and (15), the score matching objective function in Eq. (13) is simplified to the following objective function:

(16) $$E_{p(\sigma )}E_{p(\tilde{x}|x)p_{\text{data}}(x)}\left(\lambda (\sigma ,\tilde{x}-x){∥s_{\theta }(\tilde{x})+\frac{\tilde{x}-x}{\sigma^{2}}∥}_{2}^{2}\right)$$

Using deep learning techniques, the neural network $s_{\theta }(\tilde{x})$ can be trained on an image dataset to establish mixture score function.

### Dataset

We used CT images in NIH-AAPM-Mayo Clinic CT Grand Challenge dataset to train the mixture score function. The dataset consists of 2,378 CT images from 10 patients with a slice thickness of 3mm. We randomly divided the dataset into a training dataset and a test dataset. The training dataset contains 1,923 images from 8 patients, and the test dataset has 455 images from the remaining 2 patients.

### Neural Network

**Figure 6.**
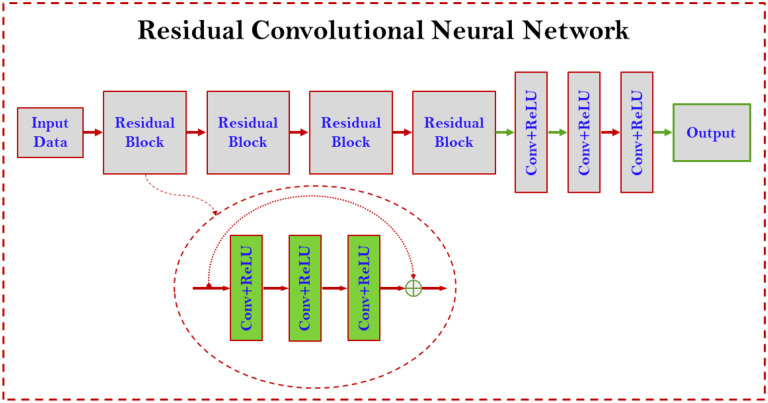
Residual neural network architecture

The standard training process was performed for the training, validation, and testing. We established a ResNet neural network to learn the mixture score function (MSF) from CT image dataset based on Eq. (16). ResNet has four residual blocks, followed by a convolution layer with 16 filters of 3×3 kernels, a convolution layer with 8 filters of 3×3 kernels, and finally a convolution layer with a single filter of 3×3 kernels to generate one feature map as the output. The first two residual blocks each have three sequential convolutional layers with 32 filters of 5×5 kernels and a residual connection. The last two residual blocks each have three sequential convolutional layers with 32 filters of 3×3 kernels and a residual connection. Every convolution layer is followed by a ReLU activation function, as shown in Figure 6.

### Data Training

Noise levels of Gaussian mixture parameters in Eq. (16) were set to: 0.00, 0.01, 0.02, 0.03, 0.04, 0.05, 0.06, 0.07, 0.08, 0.09, 0.10, which corresponding probability distribution was the binomial distribution with 10 trials and the probability of success 0.5: 0.0010, 0.0098, 0.0439, 0.1172, 0.2051, 0.2461, 0.2051, 0.1172, 0.0439, 0.0098, 0.0010. The parameters of the convolution kernels were randomly initialized by a Gaussian distribution with a mean of zero and a standard deviation of 0.01. Using image patches of 32×32, ResNet is trained to optimize the kernels in the convolutional layers by minimizing the loss function according to equation (16) on a training dataset. The training procedure was programmed in Pytorch on a PC computer with an NVIDIA Titan XP GPU of 12 GB memory. The optimization was conducted using the ADAM algorithm with the exponential decay rate $\beta_{1}=0.9$ for the first moment estimates and $\beta_{2}=0.999$ for the second-moment estimates. The training proceeded at a learning rate of 10^−4^ over 1,000 epochs, and took approximately 24 hours. This training process showed excellent convergence and stability. The trained ResNet established the mixture score function for image reconstruction, which was used in the iterative formula Eq. (8) of the Methods Section.

## Figures and Tables

**Table 1.** Image reconstruction accuracy comparisons

	Metric	FBP	SART+TV	Score function	MSF
*Case 1*	*SSIM*	*0.8183±0.0265*	*0.5647±0.039*	*0.5860±0.0387*	*0.8206±0.0215*
	*PSNR*	*35.6176±2.753*	*29.0916±2.497*	*30.0964±2.772*	*36.6089±2.048*
*Case 2*	*SSIM*	*0.8812±0.0195*	*0.9291±0.027*	*0.9139±0.0338*	*0.9327±0.0235*
	*PSNR*	*37.6007±2.356*	*38.2617±1.797*	*36.4701±1.963*	*38.7518±1.947*
*Case 3*	*SSIM*	*0.9454±0.0214*	*0.8854±0.028*	*0.9083±0.0238*	*0.9612±0.0195*
	*PSNR*	*40.0317±1.956*	*38.5586±1.597*	*39.3167±1.743*	*41.8077±1.348*
